# MYH9‐related disease: Assessment of the pathogenicity of a new mutation

**DOI:** 10.1002/jha2.715

**Published:** 2023-05-17

**Authors:** Babuty Antoine, Pierre Boisseau, Nicolas Drillaud, Marion Eveillard, Marc Fouassier

**Affiliations:** ^1^ Nantes Université CHU Nantes, Service d'Hématologie Biologique Nantes France; ^2^ Nantes Université CHU Nantes Centre de Ressource et de Compétence—Maladies Hémorragiques Constitutionnelles Nantes France; ^3^ Nantes Université CNRS 6286 UFR Sciences et Techniques 2 Nantes France; ^4^ Nantes Université CHU Nantes Service de Génétique Médicale Nantes France; ^5^ Nantes Université INSERM UMR 1307 CNRS 6075 Université d'Angers CRCI2NA Nantes France

**Keywords:** genetic, immunofluorescence, macrothrombocytopenia, MYH9‐related disease

1

A 19‐year‐old woman was addressed to the Haemophilia Treatment Center in august 2022 due to thrombocytopenia on complete blood count (CBC). Medical examination did not reveal any signs of bleeding on a daily basis, and she experienced appendectomy and avulsion of deciduous teeth without bleeding. ISTH bleeding assessment tool was 0. There was no family history of bleeding or thrombocytopenia. CBC performed in the laboratory on XN10 analyzer (Sysmex) disclosed a thrombocytopenia, (66 × 10^9^·L^−1^) by fluorocytometry. Mean platelet volume was not assessable due to platelet anisocytosis. Furthermore, immature platelet fraction was dramatically increased at 47.7%. Routine coagulation tests (PT, APTT, fibrinogen activity) were normal, and there was no von Willebrand Factor deficiency. Platelet aggregation assay revealed no abnormality. Glycoprotein receptors GpIb and GpIIbIIIa were present (GpIb 30800 and GpIIbIIIa 46437 receptors per platelet, normal values of respectively 27000–49000 and 37000–65000). Other common causes of thrombocytopenia were excluded: platelet antibodies detection by flow cytometry or by monoclonal antibody immobilized platelet antigen assay (MAIPA kit, apDia) were negative, thyroid stimulating hormone was normal, antinuclear antibodies and viral serologies for Hepatitis B virus, Hepatitis C virus and human immunodeficiency virus were negative, and there was no lupus anticoagulant.

Examination of the peripheral blood smear showed abnormal neutrophils with typical Döhle‐like inclusions in almost 100% in the neutrophils (Figure 1A). A strong platelets anisocytosis was observed with presence of macroplatelets and giant platelets (Figure 1B). Immunofluorecence labelling for non‐muscle myosin heavy chain IIA (NMHC‐IIA) protein showed small aggregates of the protein in granulocytes compared to normal control (Figure 1C,D).

**FIGURE 1:**
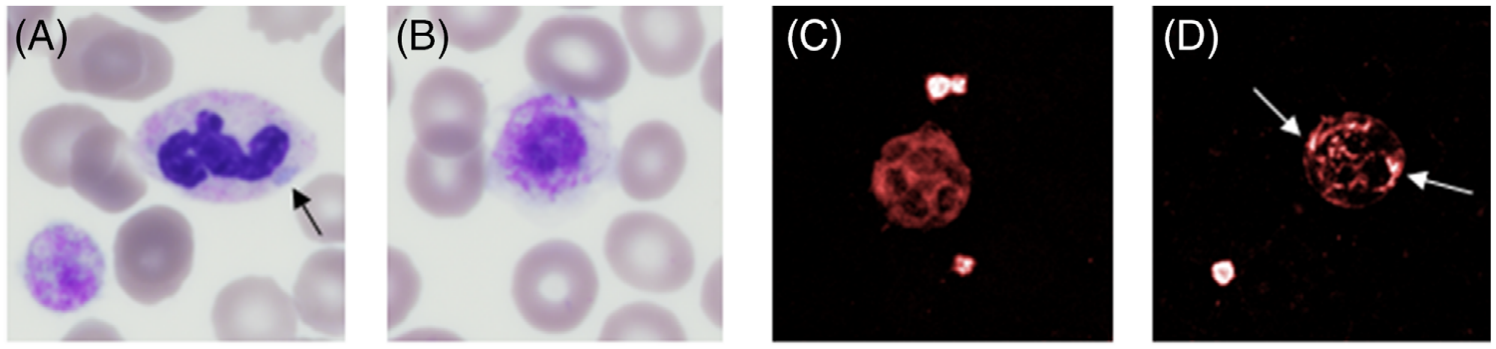
Giant platelets and non‐muscle myosin heavy chain IIA (NMHC‐IIA) inclusions in granulocytes visualized by optic microscopy and immunofluorescence. Peripheral blood films showing small inclusion in the granulocyte, consisting of non‐musclemyosin heavy chain IIA, stain light blue (A) (black arrows) and giant platelet with normal granular labelling (B), May Grünwald Giemsa stain, ×50 magnification. Small inclusions are visualized by immunofluorescence . Normal control for red labelling of non‐muscle heavy chain IIA (C) and inclusion bodies in granulocytes (white arrows, D). Operetta^®^ CLS™ high content analysis system—PerkinElmer, ×63 magnification.

**FIGURE 2:**
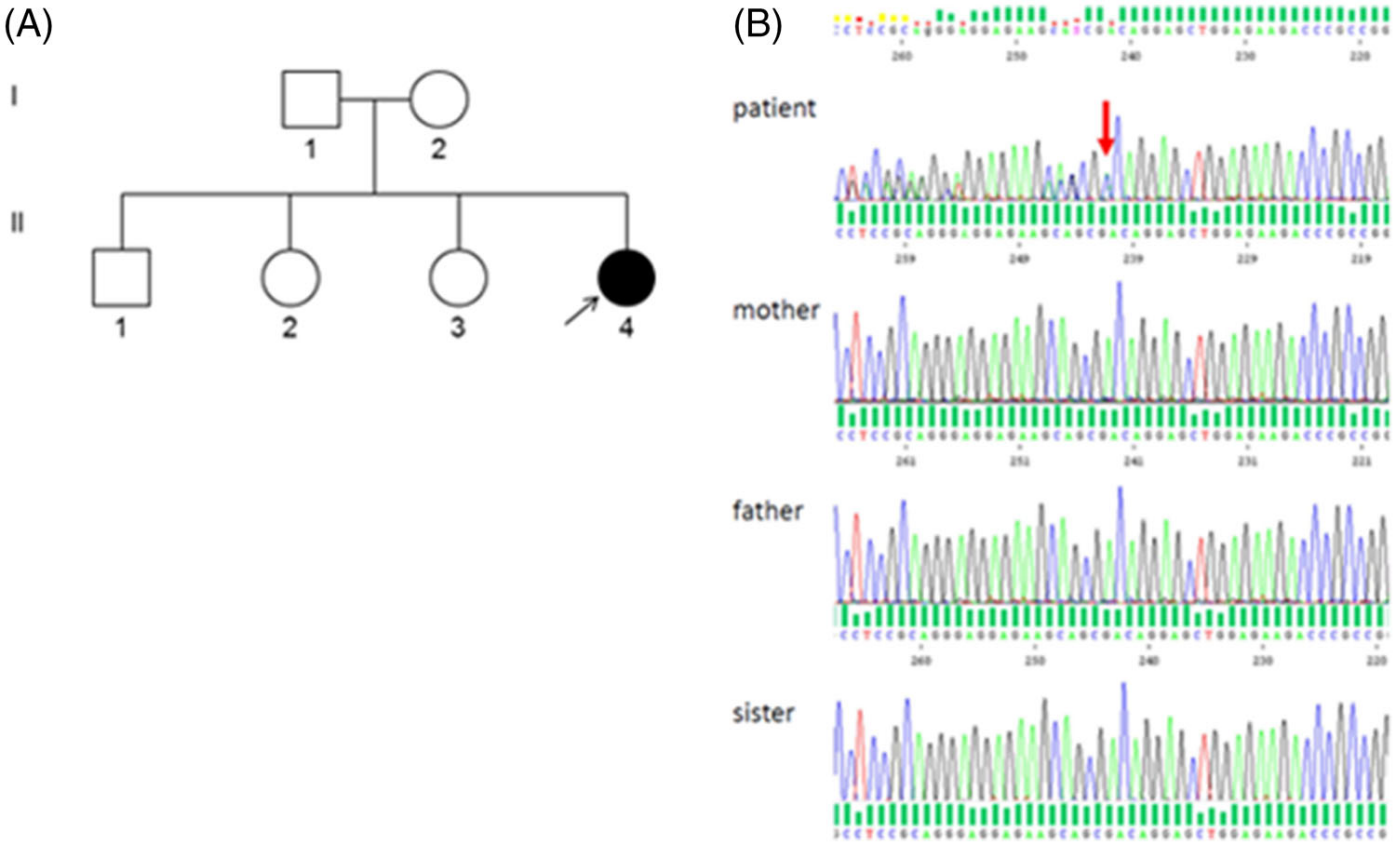
Family tree and direct sequencing of the proband and her family. (A) Family tree of the patient (black arrow). The white pattern indicates physiological condition, while the all black pattern indicate pathological condition. (B) The results of direct sequencing of MYH9 exon 25 in the proband and her family. The proband was heterozygous for c.3131_3151dup p.(Gln1044_Arg1050dup) variant.

To confirm diagnosis, we performed a gene test for MYH9‐related disease. Sequencing analysis revealed a novel c.3131_3151dup p.(Gln1044_Arg1050dup) duplication in exon 25 of MYH9 gene. This sequence variation was not found in her parents (the true paternity was confirmed) or in her siblings who all had normal platelet count and volume (Figure 2) confirming the de novo status of this variation. The de novo status of the mutation and the functional study of the protein by immunofluorescence are two strong arguments to classify this new mutation as probably pathogenic according to the American College of Medical Genetics and Genomics classification (ACMG 4) [1].

MYH9‐related disease is a rare illness involving multiple organs such as ear, eyes, kidneys, liver and blood cells [2]. At the time of diagnosis, the patient had a normal renal function and no signs of hearing or sight loss. Mutations in exon 25 can lead to modification of the structure of the helical tail of the protein with a lower risk of extra hematological manifestations than variants affecting the globular head [3]. Indeed, variants resulting in modifications of the terminal part of the helical tail or in the non‐helical tail are normally associated with isolated macrothrombocytopenia [3]. Thrombocytopenia is also less severe in variants affecting amino acid of the helical tail compared to variants in the globular head [4].

MYH9 syndrome is the leading cause of macrothrombocytopenia. The presence of Döhle‐like inclusions in granulocytes confirmed by immunofluorescence analysis is a pathognomonic sign of this disorder [5]. However, the presence of these inclusion bodies in granulocytes is not constant. Thus, in the case of macrothrombocytopenia, genetic analysis is essential to avoid misdiagnosing MYH9‐related disease and delaying the patient's management.

## AUTHOR CONTRIBUTIONS

Antoine Babuty, Pierre Boisseau and Marion Eveillard performed the research. Antoine Babuty, Pierre Boisseau, Nicolas Drillaud, Marion Eveillard and Marc Fouassier wrote the paper.

## CONFLICT OF INTEREST STATEMENT

The authors have no conflict of interest to declare.

## FUNDING INFORMATION

This research received no specific grant from any funding agency in the public, commercial or not‐for‐profit sectors.

## ETHICS STATEMENT

According with French regulations, the patient's oral consent was obtained.

## Data Availability

The data that support the findings of this study are available from the corresponding author upon reasonable request.
